# Cell differentiation and aging accompanied by depletion of the ACE2 protein

**DOI:** 10.18632/aging.202221

**Published:** 2020-11-17

**Authors:** Eva Bártová, Soňa Legartová, Jana Krejčí, Orazio Angelo Arcidiacono

**Affiliations:** 1 Institute of Biophysics, Academy of Sciences of the Czech Republic, 612 65, Brno, Czech Republic

**Keywords:** ACE2, renin, embryonic heart, lung cancer cells, human kidney embryonic cells

## Abstract

ACE2 was observed as the cell surface receptor of the SARS-CoV-2 virus. Interestingly, we also found ACE2 positivity inside the cell nucleus. The ACE2 levels changed during cell differentiation and aging and varied in distinct cell types. We observed ACE2 depletion in the aortas of aging female mice, similarly, the aging caused ACE2 decrease in the kidneys. Compared with that in the heart, brain and kidneys, the ACE2 level was the lowest in the mouse lungs. In mice exposed to nicotine, ACE2 was not changed in olfactory bulbs but in the lungs, ACE2 was upregulated in females and downregulated in males. These observations indicate the distinct gender-dependent properties of ACE2.

Differentiation into enterocytes, and cardiomyocytes, caused ACE2 depletion. The cardiomyogenesis was accompanied by renin upregulation, delayed in HDAC1-depleted cells. In contrast, vitamin D2 decreased the renin level while ACE2 was upregulated. Together, the ACE2 level is high in non-differentiated cells. This protein is more abundant in the tissues of mouse embryos and young mice in comparison with older animals. Mostly, downregulation of ACE2 is accompanied by renin upregulation. Thus, the pathophysiology of COVID-19 disease should be further studied not only by considering the ACE2 level but also the whole renin-angiotensin system.

## INTRODUCTION

ACE cleaves angiotensin I to form angiotensin II [1]. ACE and ACE2 are considered as regulators of blood pressure. Studying the polymorphisms in these genes is demanding, considering the pathophysiology of hypertension. The insertion/deletion (I/D) polymorphism of the *ACE* gene (mapped on chromosome 17q23) and single-nucleotide polymorphism G8790A of the *ACE2* gene (mapped on chromosome Xp22) were found to be the main predispositions for systemic arterial hypertension [2]. A coregulatory function between ACE and ACE2 was found in the case of vasoconstriction and vasodilatation that proceeds in the heart and kidneys. However, in the kidneys, the levels of the ACE2 protein decreased in experimental animals with hypertension. A similar phenomenon was observed in diabetic and pregnant rats [3, 4].

Interestingly, the upregulation of ACE2 was observed in failing hearts [5], such that the *ACE2* gene and its product, the ACE2 protein, play a regulatory role in the physiology of the heart, blood vessels, and kidneys. Thus, understanding the precise function of ACE2 could help to improve the treatment of the pathophysiological states of these organs. Moreover, the ACE2 protein has multiple interacting partners involved in the renin-angiotensin system, such as renin, which plays a role in regulating not only renal functions but also blood pressure [4, 6].

High ACE2 levels are observed in patients with diabetes mellitus who are cured by ACE inhibitors and blockers of the angiotensin II type-I receptor (ARBs). The ACE2 level can also be increased by the nonsteroidal anti-inflammatory drug (NSAID) ibuprofen, as observed in patients with diabetes treated with ACE inhibitors [7]. The increased expression of the *ACE2* gene could facilitate infection with pathogenic coronaviruses such as SARS-CoV [8]. Thus, it is likely that diabetes and hypertension, cured by ACE inhibitors, are comorbidities contributing to the unfavourable progression of COVID-19 infection [9, 10].

The genome of coronaviruses encodes the following proteins: spike (S) protein, nucleocapsid (N) protein, membrane (M) protein, and the protein of the envelope (E). The S protein binds to ACE2 for viral invasion into the cell [11]. Li (2016) [12] showed that the SARS-CoV-2 virus invades the cells via a short intracellular tail, a transmembrane anchor, and a large ectodomain comprising a receptor binding S1 subunit and a subunit called membrane-fusing S2. The findings that SARS-CoV-2 enters the cells via the ACE2 receptor open an avenue for effective therapies against COVID-19 disease. Therefore, it should be tested if ACE2 antibodies could prevent SARS-CoV-2 binding to the ACE2 receptor.

Based on the data mentioned above, we studied the cellular distribution pattern and cellular levels of the ACE2 protein and renin, the main factors of the renin-angiotensin system that regulate many physiological processes. To the best of our knowledge, this approach has not been applied in any other analysis of the function of ACE2 during cell differentiation and aging. We hope that our results could help clarify the role of ACE2 in SARS-CoV-2 pathogenesis and, thus, COVID-19 progression. We analyzed the ACE2 cellular distribution and protein levels in distinct cell types, established from the lungs, kidneys, hearts, and intestine of human and mouse origin, as well as studied the changes in the ACE2 level during experimentally induced mouse cardiomyogenesis. The selected cell types and mouse heart tissue were also treated with compounds that are promising from the view of COVID-19 therapy, such as vitamin D2 and dexamethasone (DEX).

Additionally, in an animal model, we tested the effect of smoking on the ACE2 level in mouse olfactory bulbs and lungs. Based on our results, we conclude that non-differentiated cells and embryos and young individuals demonstrate the highest levels of the ACE2 protein, a characteristic that should be in understanding SARS-CoV-2 infection. This analysis should also be supplemented by additional information about the status of the renin protein. Moreover, gender-specific differences in the ACE2 levels should be evaluated considering the pathophysiology of SARS-CoV-2 infection.

## RESULTS

### Localization of the ACE2 protein in the cells

**Figure 1:**
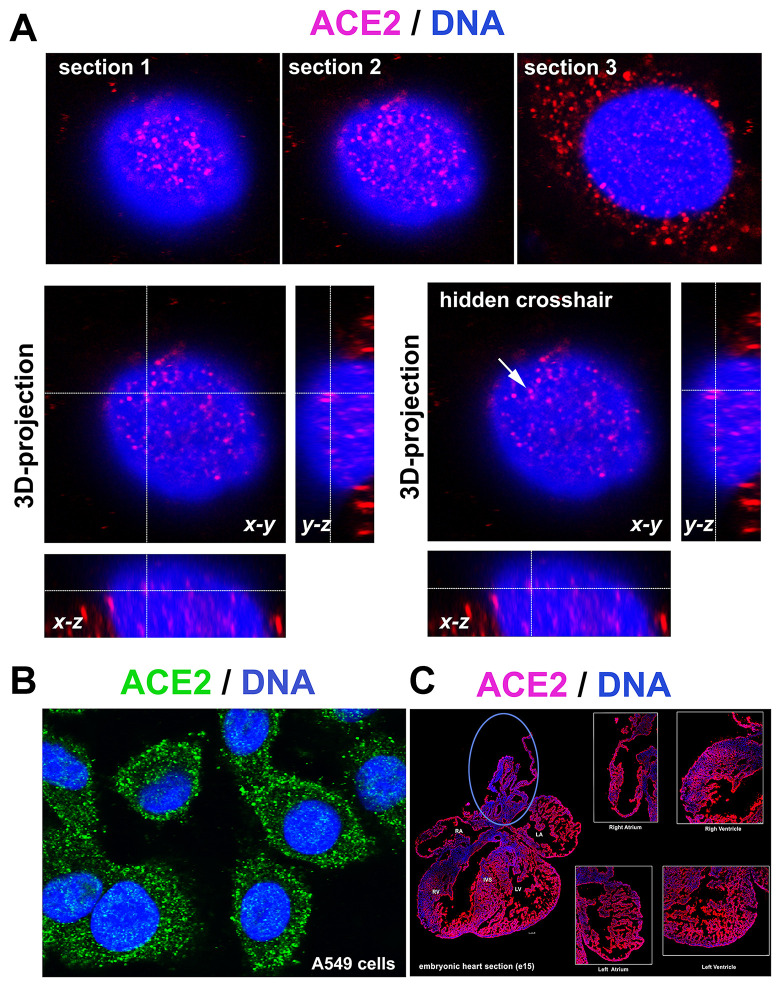
**ACE2 in lung A549 cells and mouse embryonic heart sections.** (**A**) 3D-projection of images obtained by confocal microscopy. The ACE2 protein (red fluorescence) was visualized by immunofluorescence. (**B**) ACE2 protein (green fluorescence) expression in A549 cells. (**C**) ACE2 expression in mouse heart section (red fluorescence) [see right atrium (RA), left atrium (LA), aorta (AO) and pulmonary trunk (PT), right ventriculus (RV), and left ventriculus (LV) or intraventricular septum (IVS)]. The blue ellipse shows the anatomical part associated with the aorta.

The ACE2 protein is mainly located in the cytoplasm, but we also observed fluorescence signals inside cell nuclei. 3D analysis of images from confocal microscopy confirmed the localization of the ACE2 protein inside the cell nucleus (Figure 1A). 3D reconstruction of the nuclear arrangement of the ACE2 protein is shown in lung carcinoma A549 cells, also characterized by a high density of ACE2 in the cytoplasm (Figure 1A, 1B). Additionally, we studied the density of the ACE2 protein in distinct regions of embryonic hearts (at stage e15, e.g., 15 days postconception). We analyzed the following areas of embryonic hearts: right atrium (RA), left atrium (LA), aorta (AO) and pulmonary trunk (PT), right ventriculus (RV), and left ventriculus (LV) or intraventricular septum (IVS). We observed a relatively homogeneous immunofluorescence signal for ACE2 studied in the heart sections. However, the ACE2 level in the regions around the aorta and pulmonary trunk should be considered as an exception (blue ellipse in Figure 1C).

### ACE2 level in the tissue of young and old mice

**Figure 2:**
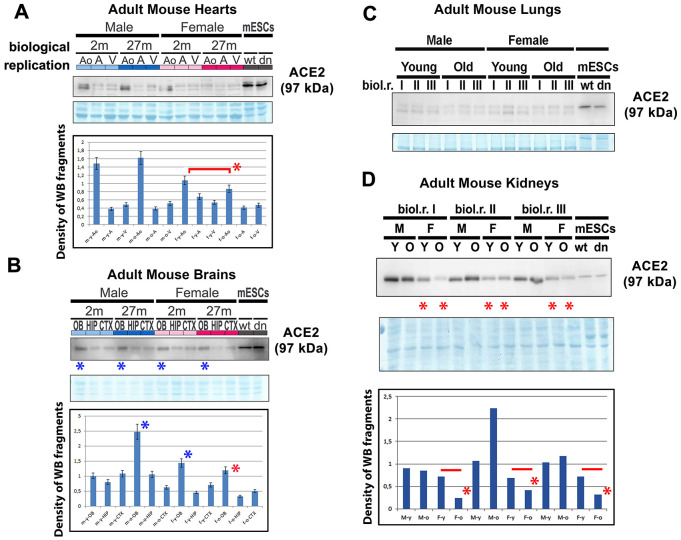
**ACE2 and renin levels in mouse hearts, brains, lungs, and kidneys isolated from young and old animals.** Adult mouse hearts were sectioned following the following anatomy: ventricular parts (V), atrium (**A**), and vessels associated with the aorta (AO). The levels of selected proteins were studied by western blotting, and the data were normalized to the total protein levels. The level of the ACE2 protein was examined in (**A**) mouse hearts, (**B**) brains, (**C**) lungs, and (**D**) kidneys isolated from young and old male and female mice. Quantification was performed using ImageJ software. Red asterisks indicate a decrease in the protein level; blue asterisks show a high ACE2 protein level compared with other samples analyzed in panel B. The nonparametric Mann–Whitney test was used for statistical analysis. Asterisks (*) indicate α=0.05.

We also studied the ACE2 protein and renin in mouse hearts isolated from young and old adult male and female animals (Figure 2A). Western blot analyses were performed with cell lysates isolated from the ventricular regions of the heart, atrium, and in the anatomical regions around the aorta (AO) and pulmonary trunk (aorta-associated vessels). Interestingly, we observed that the level of ACE2 was the highest in young female aorta regions compared with that in old female aortas. In male aortas, the level of ACE2 was identical to that compared with young and old animals (Figure 2A). When we studied the ACE2 level in mouse brains (olfactory bulbs [OB], hippocampus [HIP], cortex [CXT]), interestingly, the highest level of ACE2 was found in olfactory bulbs compared with that in hippocampi and the brain cortex (Figure 2B). These results were, in many cases, identical in both genders as well as in young and old animals (Figure 2B). In adult mouse lungs, we observed a low level of ACE2 compared with that in mouse embryonic stem cells (mESCs), which were used as reference samples (Figure 2C). However, in adult mouse kidneys, the ACE2 level was very high compared with that in mESCs and higher in male mice than in female mice of the same age (Figure 2D). Notably, old females were characterized by the lowest level of ACE2 protein in the kidneys (Figure 2D). Generally, the comparison of young and old female samples (heart AO, brain OB, HIP, CTX, and kidneys of female mice) showed a decreasing trend in the ACE2 level during aging, particularly in females (Figure 2A–2D).

### Downregulation of ACE2 in HDAC1 wt and HDAC dn mouse embryonic stem cells undergoing differentiation into cardiomyocytes

**Figure 3:**
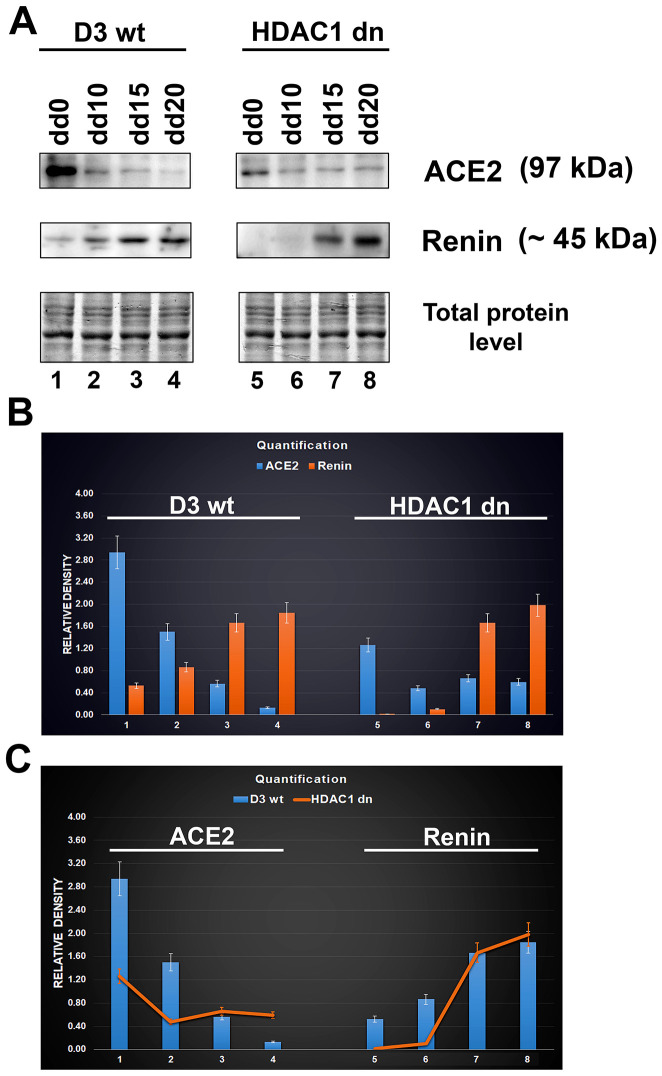
**Downregulation of ACE2 in mESCs undergoing differentiation into cardiomyocytes.** (**A**) western blot analyses were performed in HDAC1 wt (D3 wt) and HDAC1-depleted (dn) mES cells, non-differentiated and differentiated into cardiomyocytes. The data show the levels of the ACE2 protein and renin normalized to the total protein levels. (**B**) Quantification of the protein levels, as assessed by western blotting, was performed using ImageJ software; the bar chart shows the comparison of protein levels in differentiated wt mESCs and differentiated HDAC1 dn mESCs. (**C**) The graphical illustration shows the comparison of ACE2 and renin (separately) in differentiated wt mESCs and differentiated HDAC1 dn mESCs.

In non-differentiated mESCs (D3 wt), we observed a relatively high level of the ACE2 protein. Compared with HDAC1 (histone deacetylase 1)-depleted mES cells, HDAC1 wt cells were characterized by a higher level of ACE2 (Figure 3A, 3B). Experimentally induced differentiation into cardiomyocytes in both wt and HDAC1 dn cells was accompanied by the downregulation of ACE2 but upregulation of renin (Figure 3A, 3B). According to these results, the ACE2 protein and renin likely work antagonistically (Figure 3B). Importantly, HDAC1-depleted cells were characterized by a late onset of renin upregulation that appeared during cardiomyogenesis (on the 15^th^ (dd15) and 20^th^ (dd20) days of differentiation). In HDAC1 wt mESCs, renin started to be upregulated on day 10 (dd10) of differentiation (Figure 3A–3C). Together, we summarize that HDAC1 deficiency affects the properties of both ACE2 and its interacting partner renin.

**Figure 4:**
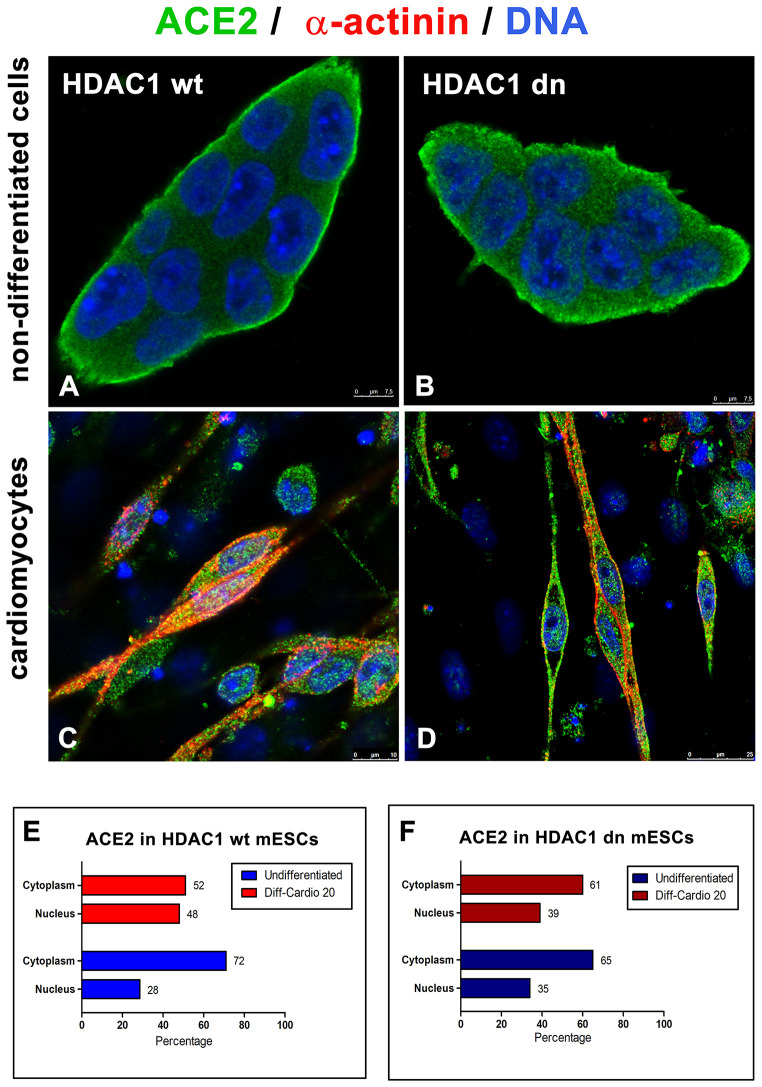
**Distribution of ACE2 (green) in wt and HDAC1-depleted mES cells, non-differentiated and differentiated into cardiomyocytes.** Immunofluorescence analyses were performed in (**A**) HDAC1 wt mESCs, (**B**) HDAC1 dn mESCs, (**C**) α-actinin (red)-positive cardiomyocytes generated from HDAC1 wt mESCs, and (**D**) α-actinin (red)-positive cardiomyocytes generated from HDAC1 dn mESCs. DAPI (blue) was used as a counterstain. (**E**) The nucleo/cytoplasmic ratio of the level of ACE2 is shown for HDAC1 wt mESCs. (**F**) The nucleo/cytoplasmic ratio of the level of ACE2 is shown for HDAC1 dn mESCs.

Additionally, in HDAC1 wt and HDAC1 dn cells, we analyzed the distribution pattern of the ACE2 protein in the cytoplasm and cell nucleus (Figure 4A–4D). In non-differentiated wt and HDAC1 dn mESCs, we observed a relatively low level of ACE2 in the cell nucleus but a high density in the nucleoplasm, particularly at the cell surface, as expected. However, cell differentiation into cardiomyocytes significantly changed the ACE2 density in the cell nucleus and ratio of the ACE2 level measured in the nucleus and cytoplasm (Figure 4E, 4F).

### The HDAC inhibitor TSA enhances the level of ACE2 in explanted embryonic hearts

**Figure 5:**
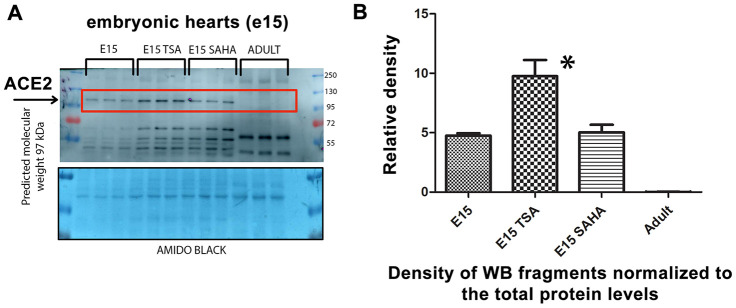
**Level of the ACE2 protein studied in explanted embryonic hearts treated by HDAC inhibitors.** (**A**) In nontreated, TSA (trichostatin A), and SAHA (suberoylanilide hydroxamic acid)-treated explanted mouse hearts at stage e15, the ACE2 protein level was analyzed. (**B**) Quantification of the protein levels, as assessed by western blotting, was performed using ImageJ software.

We analyzed explanted embryonic hearts treated with the HDAC inhibitors TSA and SAHA and observed pronounced upregulation of the ACE2 protein in TSA-treated hearts. Interestingly, embryonic hearts were characterized by a significantly higher level of the ACE2 protein than in explanted hearts from adult animals (Figure 5A, 5B). Thus, again, higher levels of ACE2 in embryonic hearts, compared with those in adult tissue, imply that ACE2 upregulation is the main characteristic of tissues of young individuals.

### Levels of the ACE2 protein in distinct cell types

**Figure 6:**
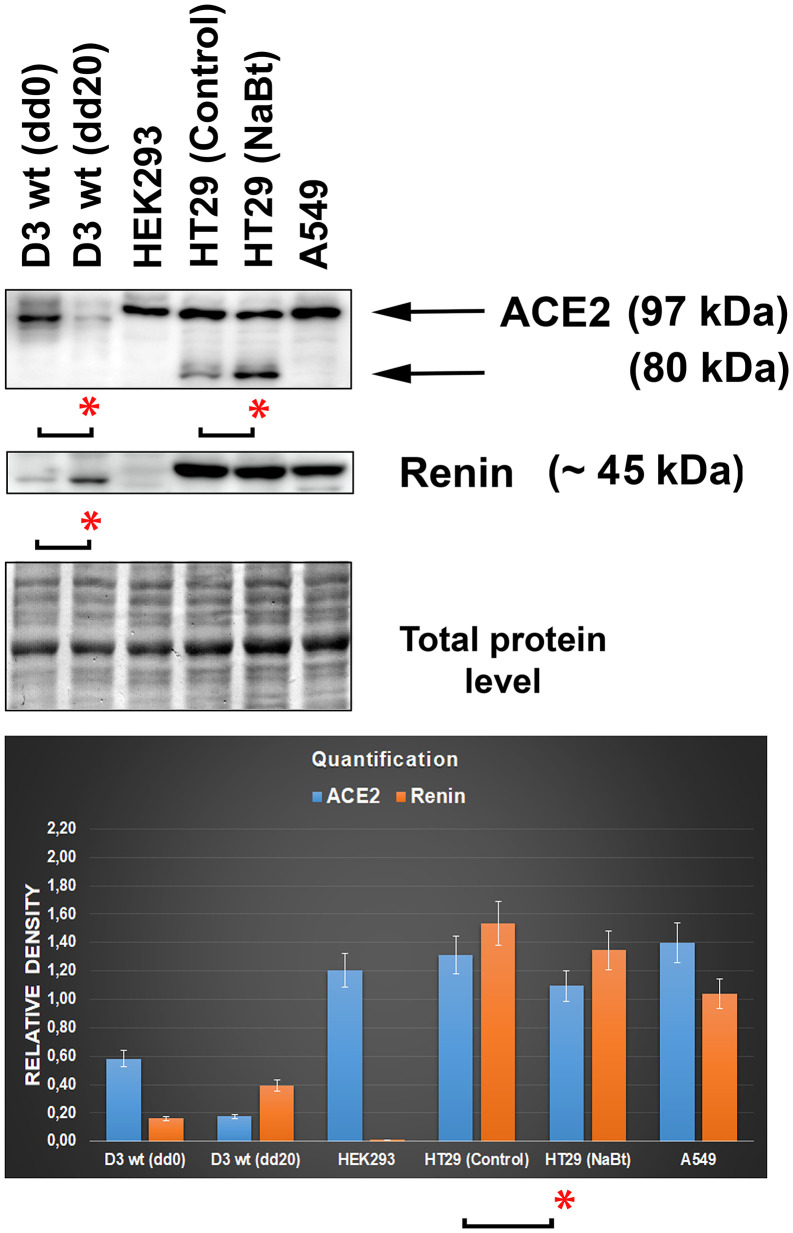
**Levels of the ACE2 and renin in distinct cell types.** The analysis was performed in mouse embryonic stem cells (mESCs) differentiated into cardiomyocytes, human embryonic kidney cells HEK293, human intestinal adenocarcinoma HT29 cells (nontreated and differentiated using the HDAC inhibitor sodium butyrate [NaBt]), and human lung adenocarcinoma A549 cells. The asterisks show a statistically significant change in the protein levels. The nonparametric Mann–Whitney test (STATISTICA software) was applied for the analysis; data with an asterisk show statistically significant differences between the tested samples (α=0.05).

By western blotting, we studied the ACE2 protein and its relationship to renin, a prominent ACE2-cooperating partner in distinct cell types (Figure 6). The levels of both ACE2 and renin were analyzed in the following cell lines representing an example of tissue preferentially affected by the SARS-CoV-2 virus: human lung adenocarcinoma A549 cells, human intestinal adenocarcinoma HT29 cells (nontreated and differentiated by the HDAC inhibitor sodium butyrate (NaBt), human embryonal kidney cells HEK293, and mouse embryonic stem cells (mESCs; line D3). In mESCs, we used the differentiation potential of these cells that can be differentiated into cardiomyocytes. We confirmed data from Figure 3A–3C that experimentally induced cardiomyogenesis is characterized by the depletion of ACE2, but the renin level was increased (Figure 6). Surprisingly, intestinal HT29 cells were characterized by the appearance of additional ACE2 fragments, whose levels were increased when the density of the main ACE2 fragment was slightly decreased by NaBt treatment initiating the differentiation of these cells into mature enterocytes (Figure 6; see differentiation protocol in [13]). Interestingly, the highest level of the renin protein occurred in intestinal cells and lung carcinoma cells but not in embryonic kidney cells HEK293 (Figure 6). This surprising fact can be explained by the observation of Shaw et al. (2002) [14], showing that widely used HEK293 cells are characterized by unexpected features of neurons; thus, these cells are not typical kidney epithelial cells.

### Lung carcinoma cells affected with vitamin D2 and other compounds promising for COVID-19 treatment.

**Figure 7:**
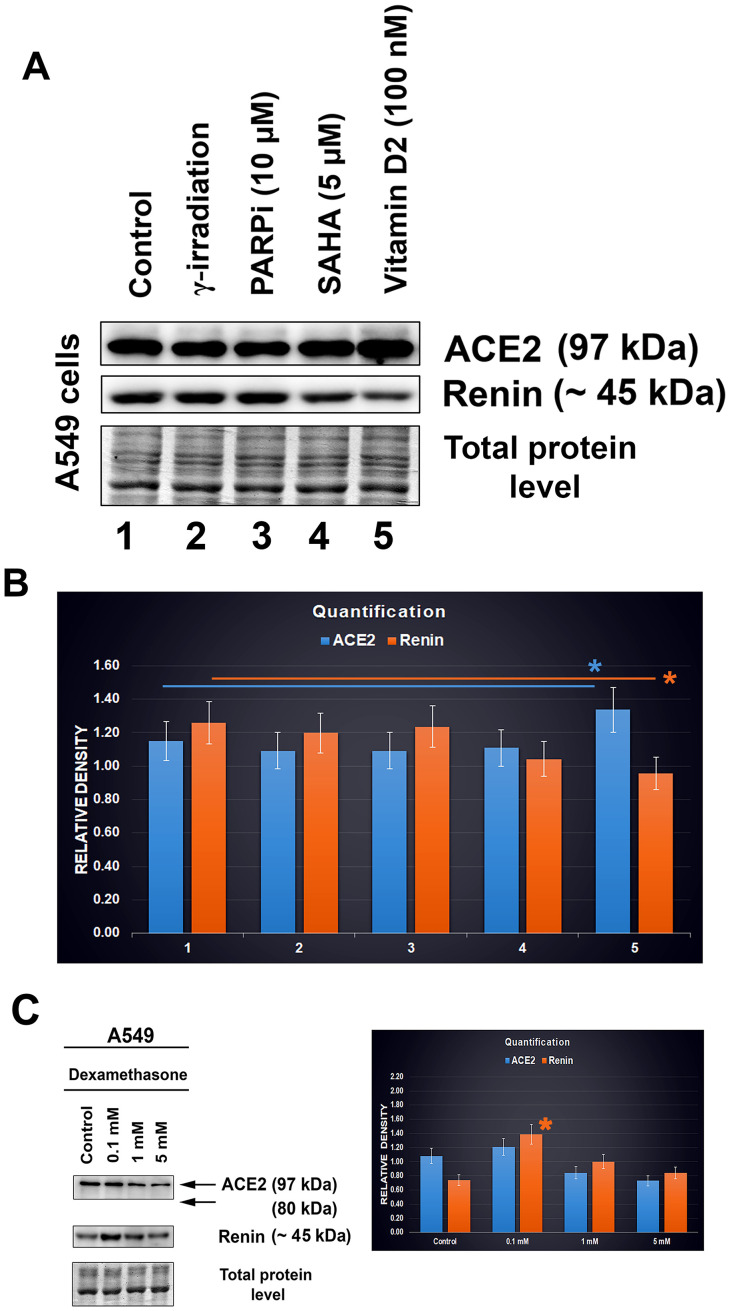
**ACE2 and renin levels after distinct treatments of A549 cells.** (**A**, **B**) The effect of the following drugs was analyzed: γ-irradiation, PARP inhibitor (olaparib), HDAC inhibitor (SAHA), and vitamin D2. © Level of ACE2 in dexamethasone-treated A549 cells. The data were normalized to the total protein levels, as shown in the bar chart. Quantification of the protein levels, as assessed by western blotting, was performed using ImageJ software. The blue asterisk indicates a slight increase in the ACE2 level, and orange asterisk shows the renin level decrease, in cells treated with vitamin D2 (panel B). The orange asterisk in panel C indicates renin upregulation caused by dexamethasone treatment. The data were analyzed by the nonparametric Mann–Whitney test.

Experiments were performed in A549 lung cancer cells treated with vitamin D2. For our analysis, we used a dose of 100 nM vitamin D2 (Figure 7A). Additionally, we also studied the effect of the PARP inhibitor olaparib, HDAC inhibitor SAHA, and γ-irradiation. After such treatment of lung carcinoma cells A549, the levels of the ACE2 protein and renin were analyzed by western blotting (Figure 7A). We found only subtle changes caused by the selected drug treatment. In particular, treatment with vitamin D2 caused a slight increase in the level of the ACE2 protein, accompanied by renin downregulation. Irradiation by γ-rays and exposure to the PARP and HDAC inhibitors did not affect the ACE2 level in A549 cells (Figure 7A, 7B). Only vitamin D2 enhanced the ACE2 level, which seems to be the benefit of this vitamin, likely reducing the severity of SARS-CoV-2 infection (Figure 7A and 7B; [15]).

We also analyzed the effect of dexamethasone (DEX). Interestingly, at the highest concentration, we observed ACE2 downregulation in A549 cells; at the low concentration, the level of ACE2 was maintained at a similar level to that in nontreated cells (Figure 7C). In this case, the renin protein was upregulated (Figure 7C, orange asterisk).

### The ACE2 level is not changed in the olfactory bulbs of laboratory animals exposed to nicotine, but changes appeared in the lungs of these animals.

**Figure 8:**
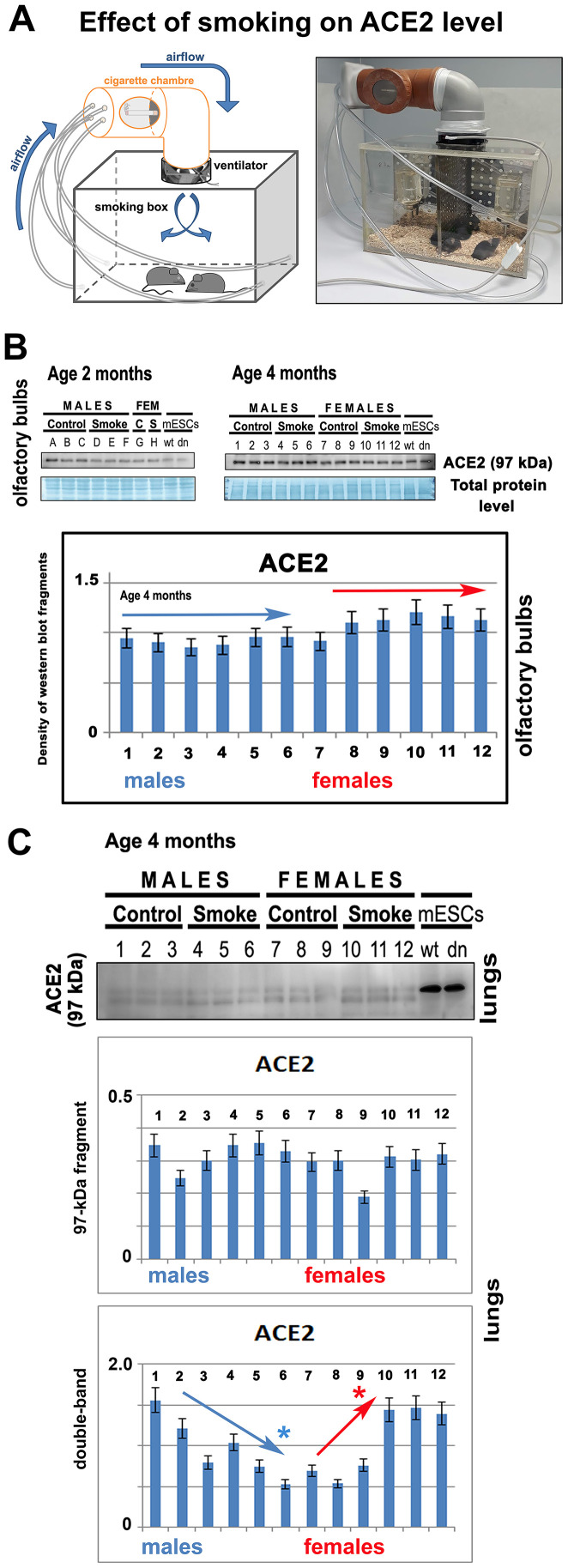
**The level of ACE2 is unchanged in the olfactory bulbs of smoking animals.** (**A**) The experiments were performed in the experimental chamber-simulated closed room with regulated ventilation. (**B**) The level of ACE2 was studied by western blotting in 2- or 4-month-old mice of both genders (smoking and nonsmoking groups). The data from 4-month-old mice were quantified by ImageJ software. The level of ACE2 was studied in explanted olfactory bulbs. (**C**) The level of ACE2 was analyzed by western blotting in the explanted lungs of mice exposed to cigarette smoke. The nonparametric Mann–Whitney test was used for statistical analysis (* indicates α=0.05).

In the experimental smoking hood, 2- or 4-month-old mice of both genders were exposed to cigarette smoke (Figure 8A). After 14 days of experiments, by western blotting, we compared the level of ACE2 in nonsmoking and smoking animals. We studied the given protein in olfactory bulbs of the mouse brain. Surprisingly, we observed no effect of smoking on the ACE2 level in olfactory bulbs. The results were identical in both 2- or 4-month-old mice (Figure 8B). In experimental animals exposed to cigarette smoke, we also studied the level of ACE2 in the lungs. Analysis of the main 97-kDa ACE2 fragment showed no statistically significant changes. However, analysis of the double band (a secondary ACE2 fragment appearing in mouse lungs; see also Figure 2C) showed the downregulation of ACE2 in male animals. In smoking female mice, there was an increased level of the ACE2 protein in the lungs (Figure 8C).

## DISCUSSION

Here, we addressed how cell differentiation, aging and selected compounds affect the levels of the ACE2 protein, a SARS-CoV-2 target. In parallel, we analyzed the level of renin, also a component of the renin-angiotensin regulatory system, that mainly regulates blood pressure. We examined whether differentiation into cardiomyocytes and enterocytes is accompanied by changes in the ACE2 and renin levels. The analysis of the ACE2 morphology showed localization of this protein not only in the cytoplasm and plasma membrane but also in the cell nucleus (Figure 1A, 1B); the highest density of ACE2 was in the nuclei of terminally differentiated cardiomyocytes (Figure 4A–4D). Similarly, Holmes et al. (2002) [16] showed that one subunit of the N-methyl-D-aspartate receptor (NMDAR) translocates from the plasma membrane to the cell nucleus. More precisely, the NR1 subunit, as a transmembrane protein, undergoes intramembrane proteolysis to release a cytosolic peptide relocated to the nucleus to modulate gene expression [16]. Additionally, Kesten et al. (2018) [17] showed that the insulin-IR (insulin receptor) complex is rearranged and moves from the plasma membrane inside the cell nucleus and then relocates to the plasma membrane or is targeted for lysosomal degradation.

In addition to morphological analysis of the ACE2 protein, we also examined changes in the ACE2 level in distinct cell types treated with selected agents, including vitamin D2. Additionally, we studied ACE2 and renin in specific cell types that are considered to be the main targets of the SARS-CoV-2 virus. Importantly, among tissues isolated from the mouse hearts, brains, lungs, and kidneys, the kidneys were characterized by the highest level of this protein (Figure 2D). At the same time, ACE2 was barely detectable in the mouse lungs (Figure 2C). However, western blotting revealed an enhanced level of ACE2 in human lung carcinoma A549 cells treated with vitamin D2 (Figure 7A, 7B). This observation agrees well with the data showing the benefit of the high ACE2 level considering SARS-CoV-2 virus biology. This virus, as its first target, affects the lung tissue that we revealed to be less ACE2 abundant. The same ACE2-decreasing trend was also observed during cardiac differentiation, which is generally pronounced during embryonic development (Figure 3A–3C). Moreover, we detected a pronounced ACE2 upregulation in wild type mouse embryonic stem cells (Figure 3A–3C, see dd0). Similarly, Valyaeva et al. (2020) [18] documented that adult stem cells can be infected by SARS-CoV-2, which could affect the self-renewal of these cells and their potential to differentiate into various cell types, the main prerequisite of tissue regeneration.

Furthermore, we studied the effect of smoking on the ACE2 level. These experiments were inspired by data published by [19]. These authors documented that chronic smoke exposure increased the level of ACE2-producing secretory cells in the respiratory tract while quitting smoking was associated with the reduction of these secretory cells and, thus, ACE2 depletion [19]. Here, we observed that short-term smoking does not increase the ACE2 level in the olfactory bulbs of female and male mice (Figure 8B); thus, changes in the ACE2 level, caused by cigarette smoke, likely appear only in the lungs, as published [19]. Our analysis of the lungs of short-term smoking animals showed ACE2 decrease in male animals, but ACE2 increase in female mice (Figure 8C). These results document gender differences when considering a response to the exposure of cigarette smoke. Although Smith et al. (2020) [19] claimed that ACE2 expression in the lungs is not correlated with age or sex, they did not study this aspect in animals exposed to cigarette smoke. They analyzed the *ACE2* gene expression in the lungs of smokers versus nonsmokers, and they saw a considerable spike in *ACE2* from one particular cell type: secretory goblet cells [19]. Thus, these secretory cells in the respiratory tract likely represent the main target of the virus. However, the question remains whether the level of ACE2 in a given tissue is determined by the number of these ACE2-highly positive cells or by the efficiency of the whole renin-angiotensin system, which can be affected by many stimuli and biological processes, including cardiac differentiation (Figure 3A–3C).

In our studies, also aging was accompanied by ACE2 depletion (Figure 2A–2D), which was mainly observed in female animals. This observation contradicts the claim that male individuals are more prone to virus infection, but the outcome is often worse in female individuals [20]. However, Jin et al. (2020) [21] showed that men and women have the same prevalence of COVID-19 disease, but men with COVID-19 have a higher risk for worse outcomes and death, independently of age.

## CONCLUSIONS

Together, our experimental data indirectly imply that the higher ACE2 levels seem to be a barrier against SARS-CoV-2 infection. An increase in the ACE2 level was mostly accompanied by renin downregulation. Thus, not only ACE2 but the whole renin-angiotensin regulatory pathway might be a target of the SARS-CoV-2 virus; therefore, at least renin function should be investigated to understand the pathophysiology of this viral infection. Our finding was also confirmed by the fact that vitamin D2 and cardiac differentiation are changing the levels of both ACE2 and renin (Figure 3A–3C, 7B). These data indicate that some dietary compounds, such as vitamins (summarized in [22]), should be further evaluated for their effect on the renin-angiotensin system, particularly in specific differentiation pathways and during aging that, according to our data, cause ACE2 downregulation. These observations led us to the conclusion that a high ACE2 level might be a barrier against SARS-CoV-2 infection. In this regard, we showed that tissues of young individuals and mouse embryonic hearts are highly abundant in ACE2 (Figure 2A, 2D and Figure 5A, 5B).

Additionally, our suggestion was based on our experiences with other studies elucidating the p53-53BP1 protein-protein interaction [23]. In this case, using FLIM-FRET technology, we showed that the highest level of p53 weakened the p53-53BP1 interaction (see TP53 mutation R273C). However, in TP53 wild-type cells (IMR90), characterized by significantly lower p53 levels as well as in mutant cells (L194F) with the same p53 level, a high degree of protein-protein interaction was found [23]. We believe that this rule could work not only for protein-protein interactions but also for the binding of the SARS-CoV-2 virus to the ACE2 receptor.

## MATERIALS AND METHODS

### Cell culture and treatment

Human lung cancer A549 cells and human adenocarcinoma HT29 cells were cultured in DMEM (Dulbecco's Modified Eagle's Medium; Sigma Aldrich, Czech Republic) supplemented with 10% fetal calf serum (FCS) (Merck, Germany). A549 lung cancer cells were treated with the PARP inhibitor olaparib (10 μM for 24 hours; #S1060, Selleckchem, Germany) and the inhibitor of histone deacetylase SAHA (5 μM for 24 hours; #10009929; Cayman Chemical Company, USA). Additionally, A549 cells were treated vitamin D2 (100 nM for 24 hours; #S4035; Selleckchem, Germany) or dexamethasone (#D4902; Sigma Aldrich, Czech Republic; 0.1 mM, 1 mM, and 5 mM (treatment was adopted from [24]).

The human intestine adenocarcinoma (HT29) cells were treated with the histone deacetylase (HDAC) inhibitor sodium butyrate (NaBt; 5 mM; #B5887; Sigma Aldrich, Czech Republic) to induce enterocytic differentiation for 48 hours (see [13]). In these experiments, we studied the effect of histone hyperacetylation and cell differentiation on the level of the ACE2 protein.

We have also studied the ACE2 level in HEK293 human embryonic kidney cells cultured in 293SFM II growth medium (#11686029, Thermo Fisher Scientific, Czech Republic). This is a serum-free, protein-free medium for the growth and transfection of the suspension-adapted human embryonic kidney (HEK293) cells. For culture, the medium was supplemented with 10% FCS (Merck, Germany).

For irradiation using cobalt-60, the cells were cultured on Petri dishes, irradiated with 5 Gy of γ-rays delivered by the Chisostat irradiation device (Chirana, Czech Republic). Next, the cells were either fixed by 4% formaldehyde for immunostaining experiments or harvested for western blotting 2 hours after γ-irradiation.

### Culture of mouse ESCs and differentiation into cardiomyocytes

The following mESC lines were studied: wild-type mESCs, D3 line (mESCs wt, wild-type), and mESCs characterized by histone deacetylase 1 (HDAC 1) deficiency (HDAC1 dn mESCs) [25]. The mouse ESCs were cultured on 0.2% gelatin-coated Petri dishes (valid for wt cells) or Matrigel-coated plastic dishes (#354277; Corning Inc., USA; valid for HDAC1 dncells). The mouse ESCs were grown in Dulbecco's Modified Eagle Medium (DMEM; Sigma Aldrich, Czech Republic) supplemented with penicillin and streptomycin, 0.1 mM nonessential amino acids, 1 ng/ml of mouse leukemia inhibitory factor (LIF), 100 μM mono-thioglycerol, and 15% fetal bovine serum (FBS). Differentiation into cardiomyocytes via the formation of embryonic bodies (EBs) was performed following [26, 27]. Cells were cultured at 37° C in a humidified atmosphere containing 5% CO_2_. Differentiation into cardiomyocytes was activated by cell seeding into ES culture media without leukemia inhibitory factor (LIF). In this step, we used the "hanging drop" method. On day 3 of culture, the EBs were placed on new plastic dishes. On day 6, the EBs were transferred to gelatin-coated culture dishes with DMEM/F12 (1:1) (#11320-033; Sigma Aldrich, Czech Republic) supplemented with insulin, transferrin, and selenium (ITS-100x; #41400-045; ThermoFisher Scientific, Czech Republic). The differentiation medium (DMEM/F12-ITS) was changed every two days. The duration of the differentiation into cardiomyocytes was up to day 20 (dd20).

### Tissue isolated from experimental animals

Explanted hearts from the mouse strain C57Bl6 were used to investigate the distribution pattern of the ACE2 protein and its interacting partner renin. The mice were kept in a pathogen-free (SPF) animal facility (see [26]). For experiments, we obtained approval from the Ethics Commission of the Ministry of Agriculture of the Czech Republic (protocol No. 48/2016). After breading, the embryos were explanted from female animals 15 days postconception (e15), and embryonic hearts were treated with HDAC inhibitors (200 nM TSA and, 16 μM SAHA) (for more details, see [28]). Except for embryonic and young (2 months), old (27 months) female and male hearts, we additionally isolated adult mouse lungs, brains, and kidneys. Using western blotting, we compared the level of the ACE2 protein in selected tissues.

### Cell culture immunostaining

Immunofluorescence was performed following [29]. Briefly, the cells were fixed in 4% formaldehyde (PFA) for 10 min at room temperature (RT) and permeabilized with 0.2% Triton X-100 (#194854; MP Biomedicals, USA) for 10 min and 0.1% saponin (#S7900; Sigma Aldrich, Czech Republic) for 12 min. After that, the dishes were washed twice in phosphate-buffered saline for 15 min. We used 1% bovine serum albumin (BSA; #A2153-506; Sigma Aldrich, Czech Republic) dissolved in 1× PBS as a blocking solution. Next, the samples were incubated in blocking solution for one hour at room temperature and then washed in 1× PBS for 15 min. For immunofluorescence analysis, the following antibodies were used: anti-ACE2 (#ab15348; Abcam, UK) and α-actinin (#A7811; Sigma Aldrich, Czech Republic). As secondary antibodies, we used the following: Alexa Fluor 594-conjugated goat anti-rabbit (#A11037; ThermoFisher Scientific, Czech Republic), Alexa Fluor 594-conjugated goat anti-mouse (#A11032; ThermoFisher Scientific, Czech Republic), Alexa Fluor 488-conjugated goat anti-rabbit (#ab150077; Abcam UK) antibodies. The negative control was considered samples incubated without primary antibodies. Cell nuclei (GC-rich sequences of DNA) were stained with 4′,6-diamidino-2-phenylindole (DAPI; Merck, Czech Republic). As a mounting medium, we used Vectashield (#H-1000, Vector Laboratories, USA).

### Heart tissue cryosectioning

Fixed mouse embryonic hearts (immersed in 30% sucrose) were frozen in embedding medium (OCT embedding matrix; Leica Microsystems, Mannheim, Germany) at −80° C. The mouse hearts were sectioned using a Leica Cryo-microtome (Leica CM 1800; Leica, Germany). The cryosections were washed in PBS, and the thickness of the sections was 13-14 μm. For immunofluorescence, we used the protocol published in [28]. The primary antibody was anti-ACE2 (#ab15348; Abcam, UK). As the secondary antibody, we used Alexa Fluor 594-conjugated goat anti-rabbit antibody (#A11037 ThermoFisher Scientific, Czech Republic). DNA was counterstained using 4′,6-diamidino-2-phenylindole (DAPI; Merck, Czech Republic), and the mounting medium was Vectashield (#H-1000, Vector Laboratories, USA).

### Exposure of laboratory animals to nicotine

In a ventilated hood, the mice were exposed to 3 cigarettes/day for 14 days. For experiments, we used both female and male animals exposed to the fumes from cigarettes P&S black (Tobacco Group PLC, 121 Winterstoke Road, Bristol BS3 2LL; 10 mg/cigarette; nicotine 0.8 mg/cigarette; carbon monoxide 10 mg/cigarette). The mice were maintained in the smoking box and exposed to nicotine and related air-pollution for 90 min each day. The experiments were performed in a closed chamber with closed airflow. Twelve 2- or 4-month-old C57BL6 mice (6 males and 6 females, in duplicated experiments) were tested. Animals of both genders were randomly divided into two experimental groups: a control group and a smoking group. Water and food were provided *ad libitum*, and no animal died during the experiments. The animals were sacrificed according to protocol No. 48/2016, and olfactory bulbs were explanted and used for analysis by western blotting.

### Tile scanning

Cryosections of whole embryonic hearts (at embryonic stage e15) were stained using appropriate antibodies, as mentioned above. For analysis, we used the "tile-scanning" mode, a tool of the Leica SP-5 confocal microscope (Leica, Germany). To acquire images, we used the HCX PL APO lambda blue 20.0× 0.7 IMM UV objective (Leica Microsystems, Germany) (see [30]).

### Laser scanning confocal microscopy

We used a Leica TCS SP8-X SMD confocal microscope (Leica Microsystems, Germany), equipped with a 63× oil objective (HCX PL APO; lambda blue) with a numerical aperture (NA) = 1.4. Image acquisition was performed using a white light laser (WLL; wavelengths of 470-670 nm in 1-nm increments) with the following parameters: 1024 *×* 1024-pixel resolution, 400 Hz, bidirectional mode, and zoom 8-12 using the Leica Application Suite (LAS X) software.

### Western blotting

Western blot analysis was performed as described previously by [31]. Cell culture or tissue samples were washed with PBS and lysed in sodium dodecyl sulfate (SDS) lysis buffer (50 × 10^3^ mol/l Tris-HCl, pH 7.5; 1% SDS; 10% glycerol). The total protein concentration was determined using the DC protein assay kit (#5000111; Bio-Rad, Czech Republic) and ELISA Reader μQuant (BioTek, USA). The proteins were separated by SDS polyacrylamide gel electrophoresis (SDS-PAGE) and transferred to polyvinylidene difluoride (PVDF) membranes. The membranes were blocked with 2% low-fat milk or 2% gelatin for one hour and then immunoblotted overnight at 4° C using the following primary antibodies against the ACE2 protein (#ab15348; Abcam, UK) and anti-renin (#PA5-102432; ThermoFisher Scientific, Czech Republic). Primary antibodies were diluted at 1:1000. As a secondary antibody, we used goat anti-rabbit IgG (#AP307P; Merck, Czech Republic; 1:2000). The western blot data were normalized to the amount of the total proteins.

### Statistical analyses and quantification of fluorescence intensity

For densitometric analysis of the western blot bands and fluorescence intensity analysis, we used ImageJ (NIH freeware) and ImageQuant TL software, respectively.

For statistical analysis, we used the Mann-Whitney U test (STATISTICA software), which is a nonparametric test of the null hypothesis that is applied for X and Y values, randomly selected from two experimental units [32]. The primary step of this analysis contains the U statistic involving the use of a symmetric real-valued function h(x,y). The following formula describes the approach:

$$U_{n}=\frac{1}{\left(\frac{n}{2}\right)}\underset{1\leq \;s\;<i}{\sum }\underset{len}{\sum }h\;\left(u_{s},u_{t}\right)$$

- (n/2) is the binomial coefficient,
- (u_t_) are independent and identically distributed variables,
- Σ = summation notation

The following two formulas are applicable for the Mann-Whitney U Test. *R* is the sum of ranks in the sample, and *n* is the number of items in the sample.

$$\begin{array}{c} U_{1}=R_{1}-\frac{n_{1}(n_{1}+1)}{2}\\ or\\ U_{2}=R_{2}-\frac{n_{2}(n_{2}+1)}{2} \end{array}$$

We ran the test at the 5% level of significance (i.e., * means α=0.05). The experiments were repeated three times; however, but in the case of smoking, we performed only two repetitions to minimize the loss of animal life.
